# A first-aid fast track channel for rescuing critically ill children with airway foreign bodies: our clinical experience

**DOI:** 10.1186/s12873-021-00482-8

**Published:** 2021-07-21

**Authors:** Yong-chao Chen, Zhi-xiong Xian, Sai-hong Han, Lan Li, Yi-shu Teng

**Affiliations:** 1 Department of Otorhinolaryngology, Shenzhen Children’s Hospital, 7019 Yitian Road, Futian District, Shenzhen, 518038 Guangdong China; 2 Department of Otorhinolaryngology, Shenzhen Children’s Hospital, China Medical University, Shenzhen, 518038 Guangdong China

**Keywords:** First-aid fast Track Channel, Foreign bodies, Trachea, Child

## Abstract

**Objective:**

To explore the role of a first-aid fast track channel in rescuing children with airway foreign bodies and to analyse and summarize the experience and lessons of the first-aid fast track channel in rescuing airway foreign bodies from patients in critical condition.

**Methods:**

We retrospectively reviewed the medical records of children with airway foreign bodies rescued by first-aid fast track channels admitted to our hospital from January 2017 to December 2020. The corresponding clinical features, treatments, and prognoses were summarized.

**Results:**

Clinical data from 21 cases of first-aid fast track channel patients were retrospectively collected, including 12 males and 9 females aged 9–18 months. Cough was the most frequently exhibited symptom (100.0%), followed by III inspiratory dyspnoea (71.4%). Regarding the location of foreign bodies, 5 cases (23.8%) had glottic foreign bodies, 10 cases (47.6%) had tracheal foreign bodies, and 6 cases (28.6%) had bilateral bronchial foreign bodies. The most common type of FB was organic. FB removal was performed by rigid bronchoscopy in every case, and there were no complications of laryngeal oedema, subcutaneous emphysema, or pneumothorax. No tracheotomy was performed in any of the children.

**Conclusion:**

The first-aid fast track channel for airway foreign bodies saves a valuable time for rescue, highlights the purpose of rescue, improves the success rate of rescue and the quality of life of children, and is of great value for the treatment of critical tracheal foreign bodies. It is necessary to regularly summarize the experience of the first-aid fast track channel of airway foreign bodies and further optimize the setting of the first-aid fast track channel.

## Introduction

As a worldwide health problem, airway foreign bodies are a commonly encountered emergency in departments of otolaryngology, especially in paediatric otolaryngology [1]. Approximately 80% of all foreign body aspirations occur in children, especially in infants below 3 years of age. Improper treatment may result in a high mortality rate, and the degree of severity depends primarily on the nature, location, and degree of blockage of foreign bodies. Common complications related to airway foreign bodies include recurrent pneumonia, emphysema, atelectasis, bronchiectasis, and even death [2]. Delayed surgical treatment may induce severe complications, such as irreversible lung injury, brain injury, and even death [3–6]. Consequently, timely and accurate diagnosis and treatment are of great significance, particularly for the prevention of complications, especially for children with airway foreign bodies in critical conditions. Our hospital has established a first-aid fast track for children with airway foreign bodies in critical condition. Different from the traditional access channel, the first-aid fast track channel has the advantages of speed, convenience and simplicity, which can greatly shorten the time for patients to see a doctor and receive treatment, and maximize the conditions for patients to receive rescue treatment in time. The first-aid fast track is composed of otolaryngology, anaesthesiology, intensive care units (ICUs), emergency department and other departments. For the first-aid fast track, the first consulting otolaryngologist may check the child and then determine the condition of the child according to the medical history, clinical symptoms, and/or auxiliary examinations, such as neck and chest CT. If the child is in critical condition, the otolaryngologist may immediately call the operating room to start the first-aid fast track. The child will be escorted to the operating room by the first consulting otolaryngologist together with the on-call anaesthesiologist and nurse in the operating room. Bronchoscopy and subsequent foreign body removal operations will be performed as the next step as soon as possible.

A retrospective analysis was carried out in this study by collecting the clinical data of 21 patients who underwent rescue through the first-aid fast track in our hospital from January 2017 to December 2020. Our study comprehensively analysed and summarized the experience and lessons of a first-aid fast track in rescuing children with airway foreign bodies in critical condition. The present study is expected to provide a potential reference for future diagnosis and treatment, especially for the screening of children with airway foreign bodies in critical condition.

## Methods

### Study design

This is a retrospective study of cases of children with airway foreign bodies treated via the first-aid fast track channel who were admitted to our hospital from January 2017 to December 2020. Every encounter has been documented, and the collected data provide information on the patients and on the intervention (e.g., demographic data, clinical presentation, radiological findings, endoscopic technique, type of FB, time elapsed between the aspiration episode and treatment, and complications). We classified dyspnoea as three forms (inspiratory dyspnoea, expiratory dyspnoea and mixed dyspnoea), and mMRC breathlessness scale is used to quantify the degree of shortness of breath— it suggests five grades (grade 0 to 4) of dyspnoea based on the circumstances in which it arises [7].

### Rescue and treatment

Due to their medical histories, clinical symptoms, physical signs and/or auxiliary examinations, the first consulting otolaryngologist ordered the first-aid fast track for the children. After calling the operating room and the supervising physician, the first consulting otolaryngologist provided the children with oxygen supplementation and delivered these paediatric patients to the operating room immediately. After arrival, the anaesthesiologist immediately initiated mask oxygen inhalation or connected the anaesthesia ventilator. The circulating nurse opened the intravenous line and monitored the respiration, heart rate, blood oxygen saturation, and ECG of the children.

Rigid bronchoscopy was the first choice for foreign bodies in the trachea [8]. All patients underwent surgery under intravenous general anaesthesia. The children were positioned with the head tilted back, and the laryngoscope was placed to expose the glottis, followed by the placement of a rigid bronchoscope of appropriate size (Karl Storz SE & Co. KG, Tuttlingen, Germany). Attention was given to the observation and aspiration of secretions when placing the laryngoscope. Meanwhile, attention was also paid to the size and shape of the foreign body intraoperatively, and the appropriate foreign body forceps were selected accordingly, and the foreign body was taken out eventually removed.

## Results

### General data

**Fig. 1:**
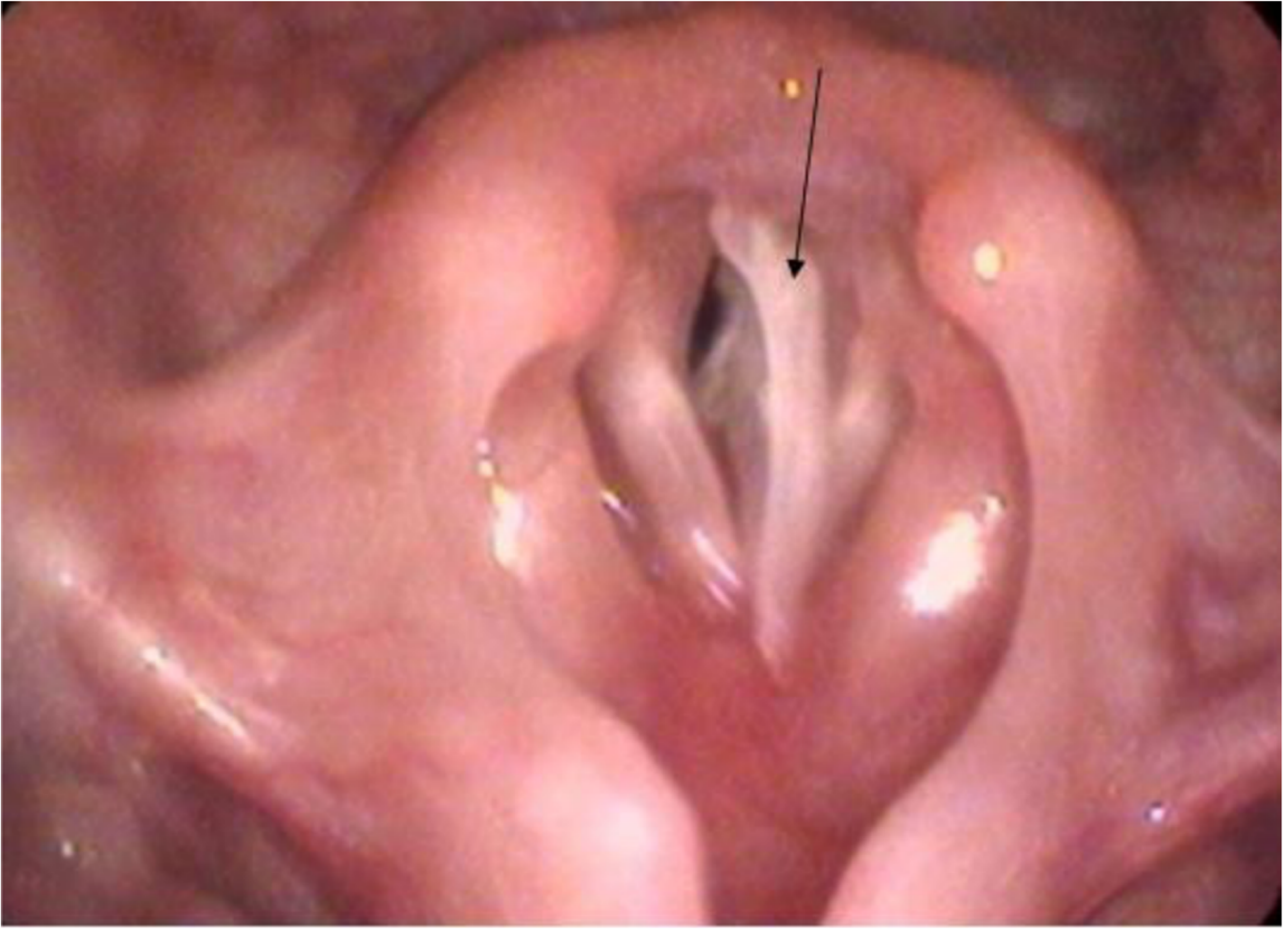
Electronic laryngoscope examination showed a foreign body in the glottis (fishbone)

**Fig. 2:**
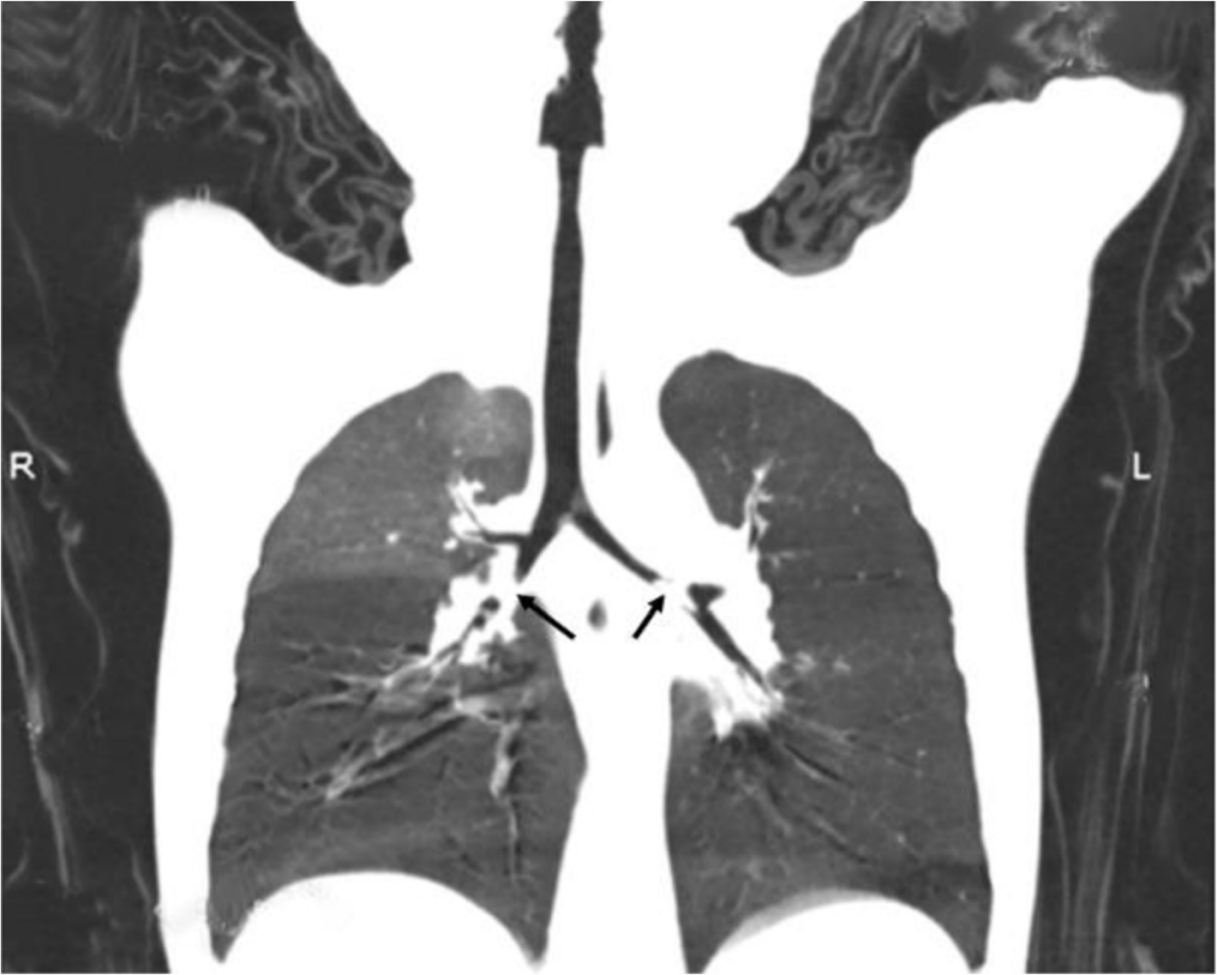
Multislice spiral CT showed a bilateral bronchial foreign body (peanut)

**Fig. 3:**
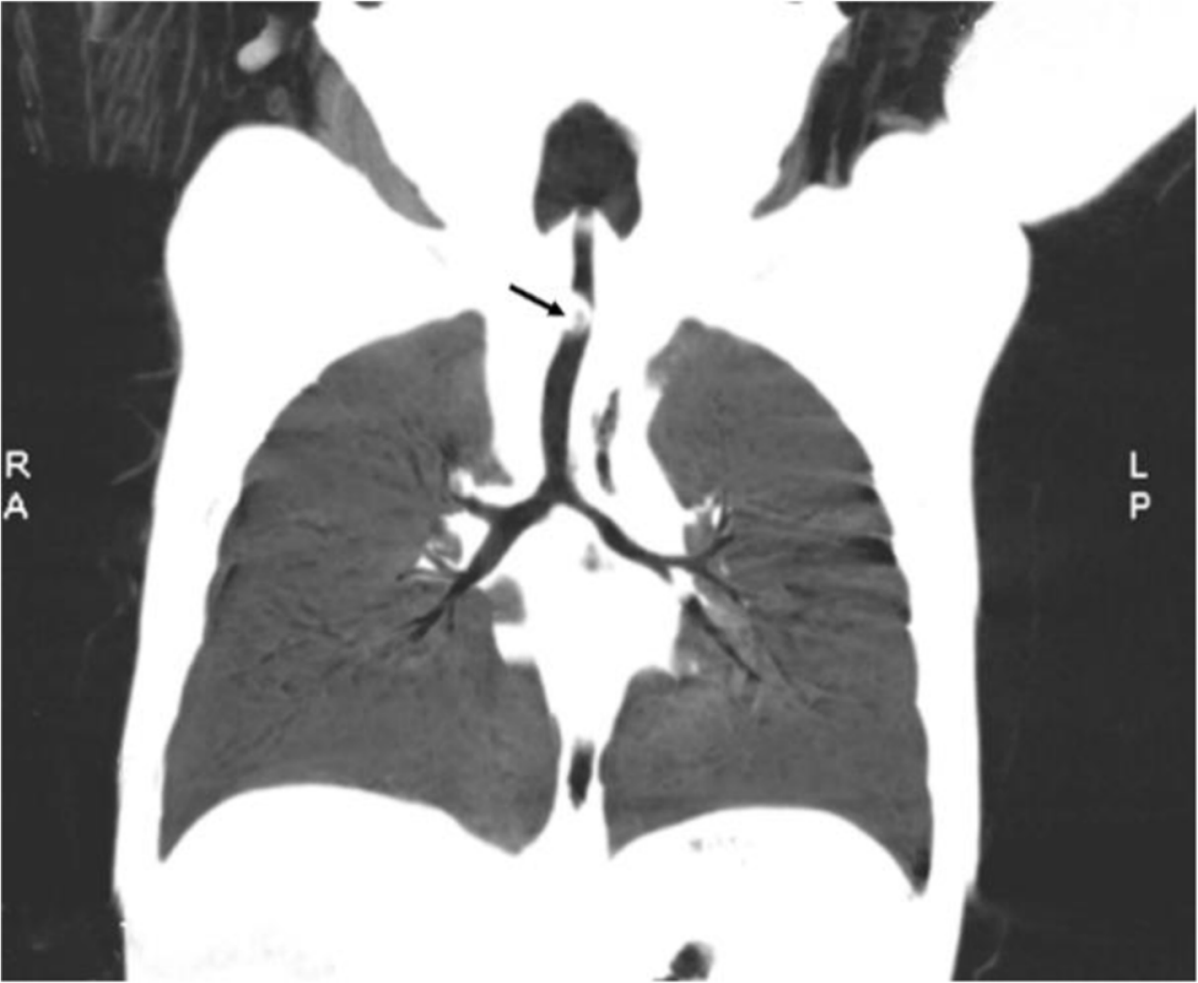
Multislice spiral CT showed an endotracheal foreign body (peanut)

**Fig. 4:**
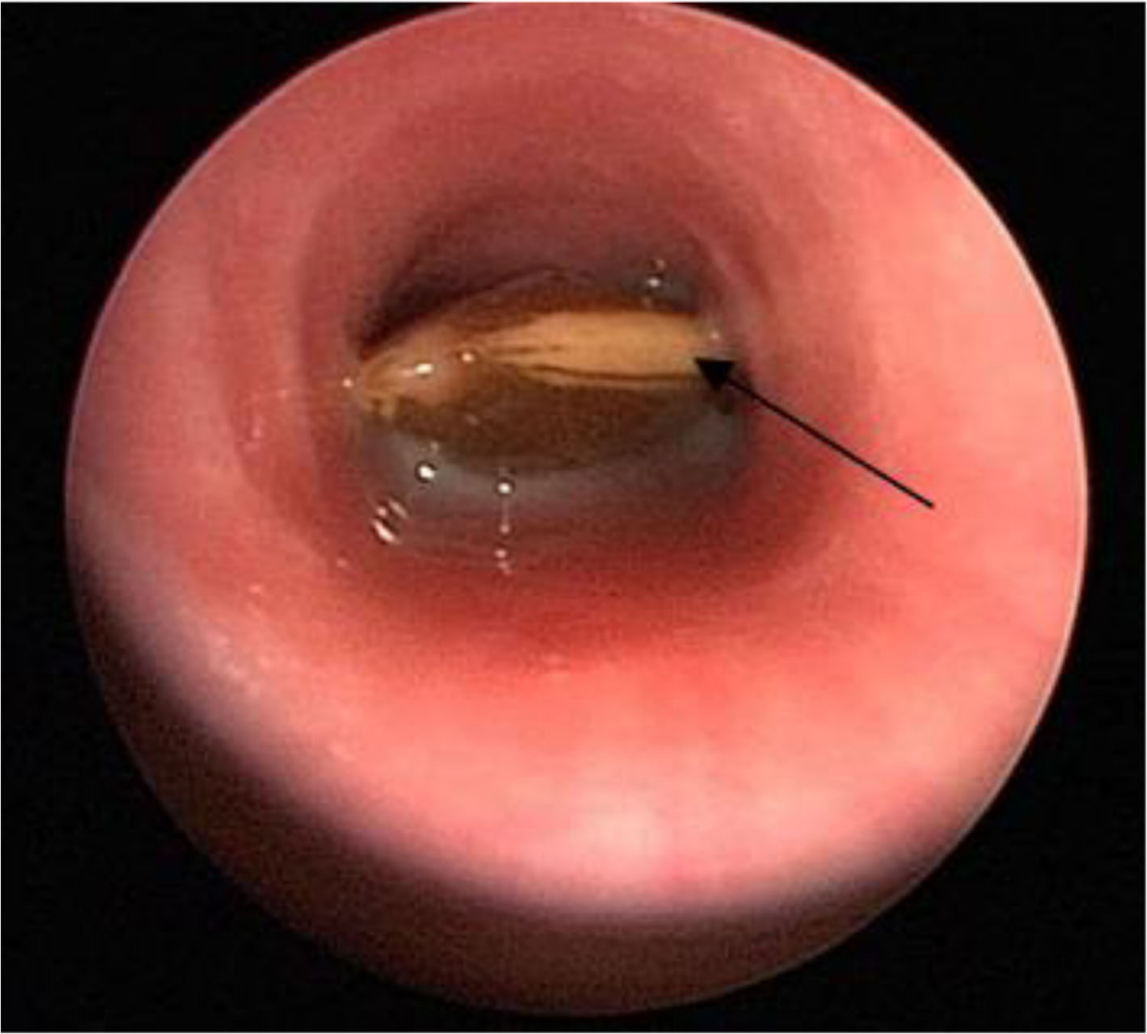
Rigid bronchoscopy showed an endotracheal foreign body (sunflower seeds)

Twenty-one cases were included in the study. The series comprised 12 boys and 9 girls, and the age was 15.0 ± 4.15 months ranging from 9 months to 18 months, and an illness duration of 1 to 72 h. The characteristics of the children rescued with the first-aid fast track channel are shown in Table 1. Regarding clinical presentation, cough was the most frequently exhibited symptom (100.0%), followed by III inspiratory dyspnoea (71.4%). There were 3 cases (14.3%) of glottic foreign bodies based on preoperative electronic laryngoscope view results, 9 cases (42.9%) of tracheal foreign bodies suggested by CT examination, and 9 cases (42.9%) of children without preoperative examination. Regarding the location of foreign bodies, 5 cases (23.8%) had glottic foreign bodies, 10 cases (47.6%) had tracheal foreign bodies, and 6 cases (28.6%) had bilateral bronchial foreign bodies. In terms of the species of foreign bodies, 4 cases were bone (Fig. 1), and of the 5 cases of glottic foreign bodies,1 case was plastic. In the other 16 cases of tracheal foreign bodies or bilateral bronchial foreign bodies, the most frequently aspirated foreign bodies belonged to the organic type, mostly peanuts (8/16, 50%, Figs. 2 and 3) and sunflower seeds (4/16, 25%, Fig. 4). In this group of children, the foreign body was removed successfully during the first operation in all cases, and there were no complications of laryngeal oedema, subcutaneous emphysema, or pneumothorax. No tracheotomy was performed in any of the children.

**Table 1:** Characteristics of children with airway foreign body rescued by the first-aid fast track channel (*n* = 21)

Characteristic	Number	Percent (%)
1. Gender		
Boys	12	57.1
Girls	9	42.9
2. Age (months)		
< 12	4	19.0
12–15^a^	7	33.33
15–18^a^	10	47.62
≥ 18	0	0
3. Clinical symptoms		
Cough	21	100.0
III inspiratory dyspnoea	15	71.4
Mixed dyspnoea	4	19.0
Assisted respiration	2	9.5
Hypoxia symptoms^b^	3	14.3
4. Preoperative examination of foreign bodies found		
Electronic laryngoscope	3	14.3
CT	9	42.9
Without preoperative examination	9	42.9
5. The location of foreign bodies		
Glottic foreign bodies	5	23.8
Tracheal foreign bodies	10	47.6
Bilateral bronchial foreign bodies	6	28.6
6. The species of foreign bodies		
Bone	4	19.0
Plastic	1	4.8
Apple	1	4.8
Peanut	8	38.1
Sunflower seeds	4	19.0
Pistachios	3	14.3

^a^ ≥ minimum, < maximum

^b^mental depression, blurred or lost consciousness, pale face, irregular heart rate, thready and rapid pulse

All 21 cases under critical condition were monitored in the PICU after tracheal intubation. One child suffered from sudden asphyxia when transferred to the operating table. Blood oxygenation failed to be maintained by mask ventilation managed by the anaesthesiologist, which decreased to an SpO_2_ of 20%, and the heart rate decreased to 30 beats/min. The anaesthesiologist entered the bronchoscope urgently while pressing the thorax and removed the foreign body, resulting in the recovery of blood oxygenation and the return of heart rate to the normal range. Another two children had a history of asphyxia rescue in another hospital and were sent to our hospital by ambulance after tracheal intubation and cardiopulmonary resuscitation. The two children could not recover despite having the foreign body removed, and the families withdrew care due to prolonged brain hypoxia and deep coma. The other 19 cases were cured and discharged from the hospital.

### Analyses of typical cases

#### Case 1

A 16-month-old female paediatric patient was admitted to the hospital “6 hours after asphyxia and 2 hours after cardiopulmonary resuscitation”. In 11:00 am, the child developed cough, cyanosis, dysphoria, and crying after eating pistachios, with a slight three concave sign. At the outside hospital, blood oxygen saturation could not be maintained, the heart rate gradually slowed to 20 beats/min, the pupils dilated to 5–6 mm, and the pupillary reaction to light was absent. In 1:00 pm, the child was given tracheal intubation and chest compression combined with other emergency treatment. Blood oxygen saturation was maintained at approximately 97%. For further diagnosis and treatment, the child was transferred to our hospital by ambulance. The child was transported to the emergency room of our hospital intubated with a heart rate of 160 beats/min, blood oxygen saturation of 95%, weak and bilateral respiratory murmur by auscultation, moist rales in both lungs, and red foam-like secretions in the endotracheal tube. The possibility of airway foreign bodies was considered in accordance with the medical history of the child and the results of physical examination. A first-aid fast track was established after obtaining informed consent from the parents. Following extubation, a rigid bronchoscope was placed during the operation, 3 pistachios were removed on the right side, and a piece of pistachio shell was removed from the left lung. The patient was monitored in the PICU with tracheal intubation. After the operation, the patient was in deep coma with no spontaneous breathing triggering, no cough reflex, and great fluctuation in blood oxygen. Brain CT showed diffuse brain oedema, and the space around the brain stem disappeared. After careful consideration, the family members withdrew care.

#### Case 2

An 18-month-old male patient was admitted to the hospital due to “cough with panting for 2 hours after eating peanuts”. Two hours prior, the child had severe cough due to a fall when eating peanuts with wheezing and no cyanosis; symptoms improved 2 min later. The parents took the child to our hospital for medical examination. Examination showed a ruddy face, no cyanosis of lips, three concave sign, a respiratory rate of 35 breaths/min, a heart rate of 120 beats/min, thick breath sounds in both lungs, slight weakness on the right side, and a few wheezes. To make a definite diagnosis, CT of the neck and chest was performed. However, when waiting for the examination, the child developed vomiting and lethargy and failed to improve prior to CT examination. Physical examination showed depressed mental status, pale face, a shallow and frequent breathing pattern, no obvious three concave sign, weak breath sounds on both sides, a few wheezes, and blood oxygen saturation of 75%. The possibility of foreign bodies in bilateral bronchi was considered, and a first-aid fast track was urgently established. Laryngoscopy was used to expose the glottis under anaesthesia, and gastric juice, mucus, and several pieces of broken peanut were found in the throat. After the placement of the rigid bronchoscope, it was discovered that gastric mucus was found in the trachea, and several pieces of peanuts were obstructing the bronchi bilaterally. The operation was successful. After the operation, the patient was transferred to the PICU with tracheal intubation for monitoring. The patient recovered normally and was discharged from the hospital.

#### Case 3

A 10-month-old male paediatric patient was admitted to the hospital due to “cough with panting for 6 hours after eating apple”. Six hours prior, the child had a severe cough, accompanied by panting and a pale face, with the cough improving after 3 min; however the panting did not resolve. The parents took the child to our hospital for medical examination, which showed a ruddy face, no cyanosis of lips, mild three concave sign, a respiratory rate of 38 breaths/min, a heart rate of 135 beats/min, thick and slightly weak breath sounds bilaterally, and obvious wheezes. The possibility of foreign bodies in the trachea was considered according to the medical history and physical examination results, and the patient was escorted to the inpatient ward. In the process of preoperative examination, the patient cried during the blood drawing in the treatment room with sudden asphyxia and cyanosis of the complexion. Emergency endotracheal intubation was carried out with balloon-assisted respiration, and the complexion of the patient returned to normal. After the establishment of a first-aid fast track, more secretions were found in the trachea during the operation, and apple pieces were observed above the tracheae carina. After the foreign body was removed, the child was transferred to the PICU with tracheal intubation for monitoring. The patient recovered normally and was discharged from the hospital.

## Discussion

Airway foreign bodies are a common emergency in paediatric otorhinolaryngology practice and are one of the main causes of accidental death in children. Zhang Yamei et al. [9] reported that the perioperative mortality rate was 1.28% in children with airway foreign bodies. Early diagnosis and prompt surgical treatment can effectively reduce the mortality rate. Airway foreign bodies are divided into critical cases, severe cases, and common cases. Once confirmed and suspected, the airways of children with airway foreign bodies in critical condition need to be explored to remove the foreign bodies as soon as possible. The top priority is to ensure the safety of the children. In this paper, a retrospective review was carried out focusing on the clinical data of 21 patients with airway foreign bodies who received treatment using a first-aid fast track established in our department over the past 4 years. This paper is intended to summarize the experience and lessons for further guidance.

There was no missed diagnosis in the 21 enrolled cases, and the parents provided a clear medical history of foreign body inhalation. Detailed medical history collection is particularly important, and both missed diagnosis and misdiagnosis will cause harm to children or even death. The visit time of the 21 children was short, ranging from 1 h to 72 h, which was related to the obvious symptoms of these children with serious illness. Of the 21 children, 10 cases of tracheal foreign bodies and 6 cases of bilateral bronchial foreign bodies were treated within 1–6 h. Among the 5 children with glottic foreign bodies, 2 cases were treated 48 h later, which may be related to the initial symptoms of hoarseness and laryngeal stridor caused by glottic foreign bodies and the asymptotic aggravation of dyspnoea.

The first consulting doctor plays an important role in evaluating children in critical conditions. For children with degree III and above inspiratory dyspnoea (), there is no difficulty in diagnosis in accordance with medical history and/or auxiliary examination, and the first-aid fast track can be quickly initiated for rescue. However, enough attention should be given to foreign bodies in the bilateral bronchi. The mortality rate of bilateral foreign bodies in bronchi was 2–3%, most of which were due to patients that could not be rescued in time and some of which were caused by improper operation or postoperative complications [10]. In this case, dyspnoea was a commonly mixed type. Meanwhile, asphyxia can occur immediately if bilateral bronchi are significantly blocked. During physical examination, there is a need to not only compare the strength of breath sounds in both lungs but also pay attention to whether there is a reduction in breath sounds bilaterally. Patients with the same degree of bilateral blockage may develop emphysema or have the same transmittance of both lungs [11]. In Case 1 and Case 2 reported in this study, the postoperative diagnosis was bilateral foreign body in bronchi with mixed three concave sign, which was mixed dyspnoea, and the appearance of low breath sounds in auscultation of both lungs. In the chest X-ray film of Case 1, the transmittance of both lungs was the same, and there was no mediastinal shift. In addition, the successful rescue of Case 2 suggested that rapid and effective treatment can avoid serious complications and even death. It is very important for the first consulting doctor to evaluate the patient’s condition. In the case of severe conditions, the first-aid fast track should be opened immediately for rapid and effective treatment to avoid serious complications.

In terms of the influence of the type of foreign body on the disease, there are many types of foreign bodies that can become lodged in children’s airways, including plants, animals, mineral compounds, etc., particularly plant species, such as peanuts and melon seeds. In general, the free fatty acids of plants may produce greater stimulation to the airway, leading to mucosal congestion and swelling, increased secretion, aggravated airway obstruction and worsened dyspnoea, thus increasing the difficulty of operation [12, 13]. Cases 1, 2 and 3 were all vegetable foreign bodies, which greatly irritated the airway. Despite a short inhalation duration of the foreign body, there were significant secretions in the airway during the operation, which accelerated the development of the disease. Furthermore, among the 5 cases of glottic foreign bodies, bone and plastic pieces produced a larger physical stimulation to the airway rather than a direct chemical stimulation.

Concerning the necessity of imaging examination, the detection rate of MSCT for tracheal foreign bodies in children is significantly higher than that of X-ray examination, and the former approach can clearly show the position, shape and size of foreign bodies. Therefore, computed tomography displays superiority in paediatric tracheal examination [14]. Both chest X-ray and chest fluoroscopy can show the presence and location of foreign bodies [15]. However, children need to be in a quiet state during CT examination. For children who cannot sleep, chloral hydrate (0.5 ml/kg) should be given for sedation; however, this is not suitable for children with obvious dyspnoea. Additionally, when children cough or move violently during chloral hydrate sedation, the foreign body may also change in position or from unilateral to bilateral, resulting in the aggravation of dyspnoea. In Case 2, gastric juice-like mucus and several pieces of broken peanuts were found in the throat when the glottis was exposed by a laryngoscope under anaesthesia, with the detection of gastric juice-like mucus in the trachea through a rigid bronchoscope. The patient cried during sedation by an enema of chloral hydrate, resulting in aspiration after vomiting, which aggravated dyspnoea. In this regard, it was necessary to judge the condition of the child based on the medical history, symptoms and signs of foreign body inhalation, and it was determined that imaging examination was unnecessary. Moreover, the parents of the child should be informed to pay close attention to the breathing, complexion, and mental state during the period of waiting for examination and should not leave the hospital.

Our study suggests that foreign bodies in the trachea, bilateral bronchi, or glottis are critical cases of grade III or IV dyspnoea before surgery and should be treated urgently. Sedation, oxygen inhalation, ECG monitoring (endotracheal intubation-assisted mechanical ventilation if necessary), venous access, and a first-aid fast track should be provided immediately.

## Conclusion

The first-aid fast track for the treatment of airway foreign bodies refers to the cooperation of otolaryngology, anaesthesiology, ICU, emergency room, and other departments, reflecting the overall rescue ability of the hospital. The establishment of a first-aid fast track in children with airway foreign bodies in critical condition saves precious time for rescue, highlights the purpose of rescue, and improves the success rate of rescue and the quality of life of children. This fast track is of great significance for the treatment of paediatric airway foreign bodies in critical condition. It is necessary to constantly summarize the experience of the first-aid fast track channel of airway foreign bodies and further optimize the setting of the first-aid fast track channel.

## Data Availability

The datasets used and/or analysed during the current study are available from the corresponding author (YS.T, tys118@163.com) on reasonable request.

## Abbreviations

ICU: Intensive care unit
FB: Foreign Body
